# Regulating Biocondensates within Synthetic Cells via Segregative Phase Separation

**DOI:** 10.1021/acsnano.4c18971

**Published:** 2025-04-28

**Authors:** Chang Chen, Caroline M. Love, Christopher F. Carnahan, Ketan A. Ganar, Atul N. Parikh, Siddharth Deshpande

**Affiliations:** † Laboratory of Physical Chemistry and Soft Matter, 4508 Wageningen University & Research, 6708 WE Wageningen, The Netherlands; ‡ Department of Materials Science and Engineering, 8789 University of California, Davis, Davis, California 95616, United States; § Biophysics Graduate Group, University of California, Davis, Davis, California 95616, United States; ∥ Department of Biomedical Engineering, University of California, Davis, Davis, California 95616, United States; ⊥ Singapore Centre for Environmental Life Sciences Engineering, Nanyang Technological University, 636921 Singapore; # Institute for Digital Molecular Analytics and Science, Nanyang Technological University, 637551 Singapore

**Keywords:** synthetic cells, segregative phase separation, coacervates, liposomes, membraneless organelles, microfluidics

## Abstract

Living cells orchestrate a myriad of biological reactions within a highly complex and crowded environment. A major factor responsible for such seamless assembly is the preferential interactions between the constituent macromolecules, that can drive demixing to produce coexisting phases and thus provide dynamic intracellular compartmentalization. However, the way multiple-phase separation phenomena, occurring simultaneously within the cytoplasmic space, influence each other is still largely unknown. Here, we show that the interplay between segregative and associative phase separation within cell-mimicking confinements can lead to rich dynamics between multiple phases and the lipid boundary. Using on-chip microfluidic systems, we encapsulate the associative and segregative components and externally trigger their phase separation within cell-sized vesicles. We find that segregative phases create microdomains and tend to dictate the fate of associative components by acting as molecular recruiters, membrane-targeting agents, and initiators of condensation. The obtained multiphase architecture provides an isolated microenvironment for condensates, restricting their molecular communication as well as diffusive motion, and can further lead to global shape transformation of the confinement itself in the form of wetted, hierarchical domains at the lipid membrane. In conclusion, we propose segregative phase separation as a universal condensation regulation strategy by managing their molecular distribution, process initiation, and spatial localization, including membrane interaction. The presented interplay between the two phase separation systems suggests a distinct design principle in constructing complex synthetic cells and controlling the behavior of artificial membraneless organelles within.

## Graphical abstract

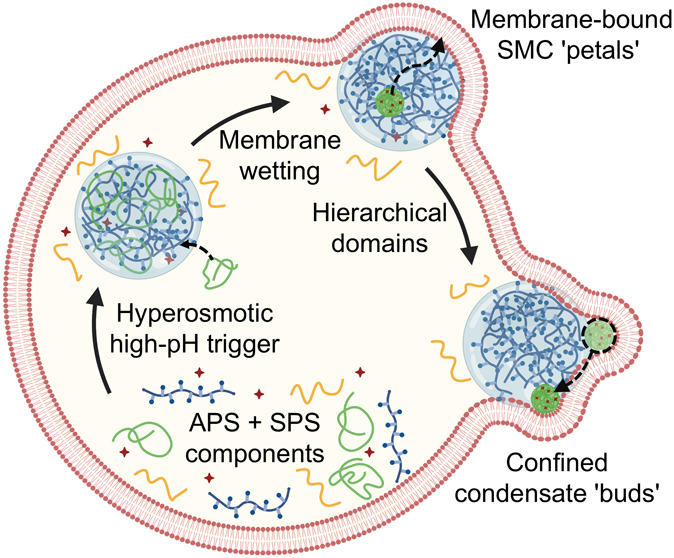

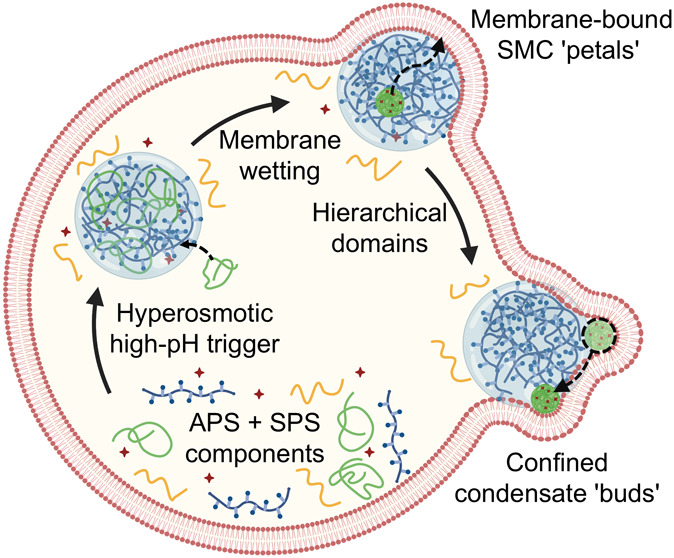

## Introduction

The interior of a living cell is an incredibly crowded and dynamic environment packed with a vast array of biopolymers and organelles. In this extraordinarily crowded aqueous space, proteins, polysaccharides, nucleic acids, and other biomolecules constantly deplete and interact with each other, creating a highly active ecosystem. To unravel the underlying principles, one fruitful approach involves constructing self-assembled, cell-mimicking systems using key biomolecules. Commencing with simple models, such as cell-sized vesicles, one can customize and engineer biological systems, leading to increasingly complex intracellular architectures.^1–3^ Both membrane-bound^4–8^ and membraneless^9–11^ modules can be utilized to achieve this, with the latter being more dynamic and gaining increasing attention.^12–14^

Within the intracellular environment, membraneless organelles^15^ serve multifaceted functions^16–18^ and often interact with cell membranes.^12^ As a result, understanding and applying model membraneless organelles is key to engineering complex synthetic cell systems, achieve basic cellular functions, and move toward specific applications.^19^ Since the groundbreaking discovery of membraneless organelles such as P granules^20^ and the nucleolus,^21^ the mechanism behind their formation, the phenomenon of liquid–liquid phase separation (LLPS), has garnered increased recognition.^20^ Based on the molecular organization within the resulting phases, LLPS can be broadly characterized as either associative phase separation (APS) or segregative phase separation (SPS). APS occurs as a result of strong intermolecular forces, such as charge-based attraction between molecules, causing them to form a condensed liquid phase, commonly referred to as a coacervate or a condensate.^22^ In contrast, SPS is driven in mixtures of polymers with distinct chemical properties, resulting in immiscible, segregated polymer-rich phases, thereby minimizing contact between them.^23^ While there is no clear evidence that SPS occurs in living cells, it could be a potent mechanism to temporally initiate and spatially localize functional condensates formed via APS, within synthetic cell systems.

In recent years, there have been active efforts to mimic cellular systems by triggering APS inside synthetic cells leading to dynamic processes, such as reversible condensation,^24^ division,^25^ intracellular trafficking,^26^ biochemical reactions,^9^ and membrane interactions.^27^ Despite these advancements, achieving precise localization of coacervates, such as to the membrane, still requires the use of specific ingredients such as charged lipids,^28^ or condensates with hydrophobic properties.^29^ An accompanying challenge is the restriction of the coacervate movement, with existing strategies still allowing them to move freely across the entire membrane surface.^29^ This nonspecific localization may not always be favorable for coacervate functionality, for example, to attain polarity or restrict physical contact with other membrane-bound entities. Last but not least, the cross-talk between condensates and the membrane’s inner leaflet requires further exploration.^30^ Similar to wetting-induced vesicle shape changes^31^ or coacervate penetration^32^ from outside, triggering coacervate-membrane interaction from inside could lead to budding- or even exocytosis-like behavior.^33^ Thus, gaining spatiotemporal control over internal coacervates is crucial for synthetic cell studies.

SPS, on the other hand, is considered to be an important tool for mimicking cellular behavior in a crowded environment. Utilizing common molecular crowders, SPS is widely applied to achieve compartmentalization within cell-mimicking confinements via temperature^34^ and osmotic triggers.^35^ The resulting LLPS can influence morphological changes in such systems including budding,^36^ tube formation,^37^ asymmetric division,^38^ as well as distribution,^39^ phase separation of membrane lipids,^23^ and even transmembrane transport.^2^ Using liposomes, prior studies have used SPS to construct polar structures,^35^ or multiple distinct subcompartments.^23^ Although APS and SPS are both well-established individually, their simultaneous and dynamic occurrence within cell-mimicking confinements remains unclear. Therefore, understanding the APS–SPS interaction and combining their advantages for bioengineering is currently lacking.

In this paper, we explore and utilize APS–SPS interactions within cell-mimicking confinements to regulate various coacervate behaviors, including molecular enrichment, membrane-targeting, restricted diffusion, and budding at lipid membranes. Using poly­(ethylene glycol) (PEG)/dextran (DEX) as the SPS system and poly-l-lysine (PLL)/adenosine triphosphate (ATP) as the APS system, we first demonstrate the recruitment of coacervates and the physicochemically restrictive environment offered by the SPS-induced membrane-free confinements (SMCs), i.e., DEX-rich domains. Then, utilizing lab-on-a-chip synthetic cell systems in the forms of double emulsions and liposomes, we investigated free as well as membrane-bound SMCs in relocating specific coacervate components (PLL) to the lumen or to the membrane. We further constructed multiphase coacervate-in-SMC architectures that could be either suspended in the lumen or distributed on the membrane in the form of micron-sized buds. When confined to the membrane by SMCs, the coacervates remained largely isolated from each other and showed a highly restricted diffusion. Overall, this generic design principle provides a broadly applicable path for mimicking and spatiotemporally controlling membraneless organelles in synthetic cells and further provides a platform for understanding the APS–SPS–membrane interplay relevant to living cells.

## Results

### SPS Physicochemically Regulates APS (Condensate) Dynamics

We utilized PEG (8 kDa) and DEX (≈10 kDa), two commonly used chemically dissimilar polymers, as model systems for SPS (Figure 1a). To facilitate subsequent experiments, we first established binodal curves for PEG/DEX using a variation of cloud-point titration for varying conditions of pH, viscosity, and buffer concentrations (see Materials and Methods for details; also see Supporting Figure 1). Regardless of the varied conditions, the system exhibited consistent binodal curves (Figure 1b), providing us with a reliable compartmentalization system in subsequent experiments (Figures 2, 3, and 4). As a model APS system, we used PLL and ATP, an extensively used molecular pair that undergoes complex coacervation via electrostatic attraction. To begin investigating the APS-SPS interactions, we assessed the tendency of PLL and ATP to get sequestered into one of the SPS phases by calculating their partition coefficients (*K*) for the two phases (see Materials and Methods for details). As can be clearly seen from Figure 1c, there is a pronounced increase in the ratio of PLL fluorescence in the DEX-rich phase over that in the PEG-rich phase (*K*_PLL_), with the values increasing significantly further with increasing concentrations of PEG or DEX. On the contrary, *K*_ATP_ exhibited a value around 1 irrespective of the PEG/DEX concentrations (see Figure 1d), indicating ATP did not show any significant preference for either phase.

**Figure 1:**
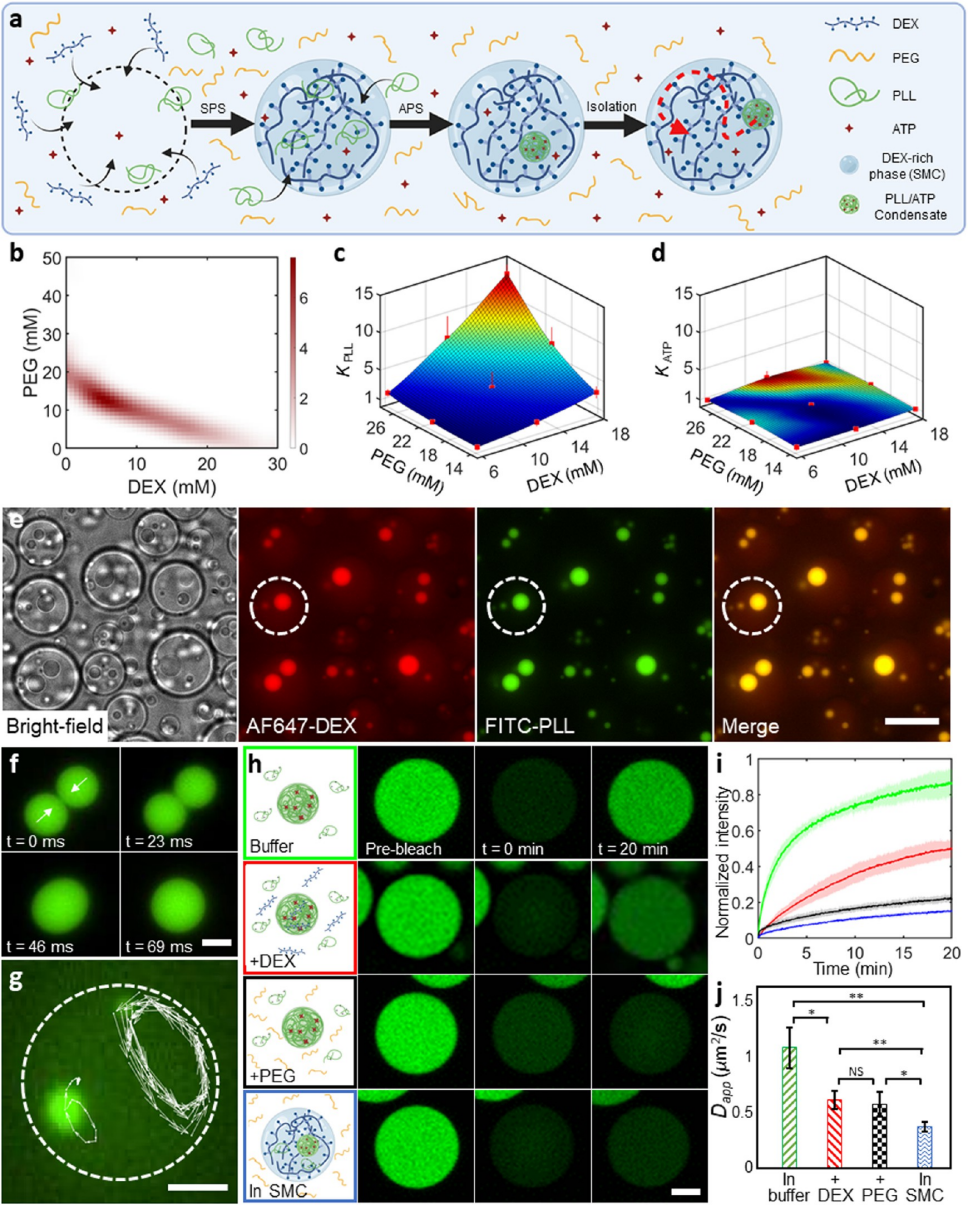
SPS domains regulate APS dynamics. (a) Conceptual sketch showing the SPS–APS interplay: SPS generates a DEX-rich domain that sequesters PLL molecules, inducing PLL/ATP coacervation and restricting coacervate motion within the SMC. (b) A combined binodal curve of PEG (8 kDa) and DEX (10 kDa), obtained by cloud-point titration, under different conditions of pH (3.9 to 9.7), viscosity (with and without glycerol), and ionic strengths (15 and 25 mM Tris-HCl buffer) used throughout this work. Regardless of the conditions, the binodals showed the same trend. The color depth corresponds to the number of data points. (c, d) Three-dimensional surface plots representing the partition coefficients (*K*) of (c) FITC-PLL and (d) cy3-ATP within the SPS system, where *K* represents the ratio of fluorescence in the DEX-rich phase over that in the PEG-rich phase. Colors represent the extent of partitioning, with red representing higher partitioning and blue representing lower partitioning. (e) Microscopy images (from left to right: bright-field, DEX fluorescence, PLL fluorescence, merged fluorescence) showing the formation and restriction of coacervates within DEX-rich domains. The dotted circle indicates the boundary of one such SPS domain. As can be seen, fluorescent DEX gets further enriched within the coacervates. Scale bar, 20 μm. (f) PLL/ATP coacervates show their usual liquid-like behavior in the form of droplet fusion within SPS domains. (g) The coacervates remain confined within the DEX-rich domains even in the presence of an external fluid flow, as shown by their confined trajectories. The dotted circle indicates the SMC boundary. (h) Time-lapse images taken by CLSM showing the fluorescence recovery of PLL (3.6 mg/mL)/ATP (4.8 mM) coacervates in varying conditions. For f–h, scale bar, 5 μm. (i) Fluorescence recovery curves of FITC-PLL after bleaching the entire PLL/ATP coacervate under varying conditions as indicated in h. (j) Corresponding diffusion coefficients of PLL after fitting the FRAP curves obtained in i. NS indicates no significant difference (*p* > 0.05); * and ** denote significant differences with *p* ≤ 0.05 and *p* ≤ 0.01, respectively. In e–g, the experiments were conducted by mixing 3 mg/mL PLL, 4 mM ATP, 12 mM DEX, and 12 mM PEG and observed by wide-field fluorescence microscopy. In (h–j), the experiments were conducted by mixing 3.6 mg/mL PLL and 4.8 mM ATP without PEG and DEX (green panel), with 12 mM DEX (red panel), with 12 mM PEG (black panel), and with 12 mM DEX and 12 mM PEG (blue panel). Data points represent mean values, with error bars and shaded areas indicating standard deviations.

**Figure 2:**
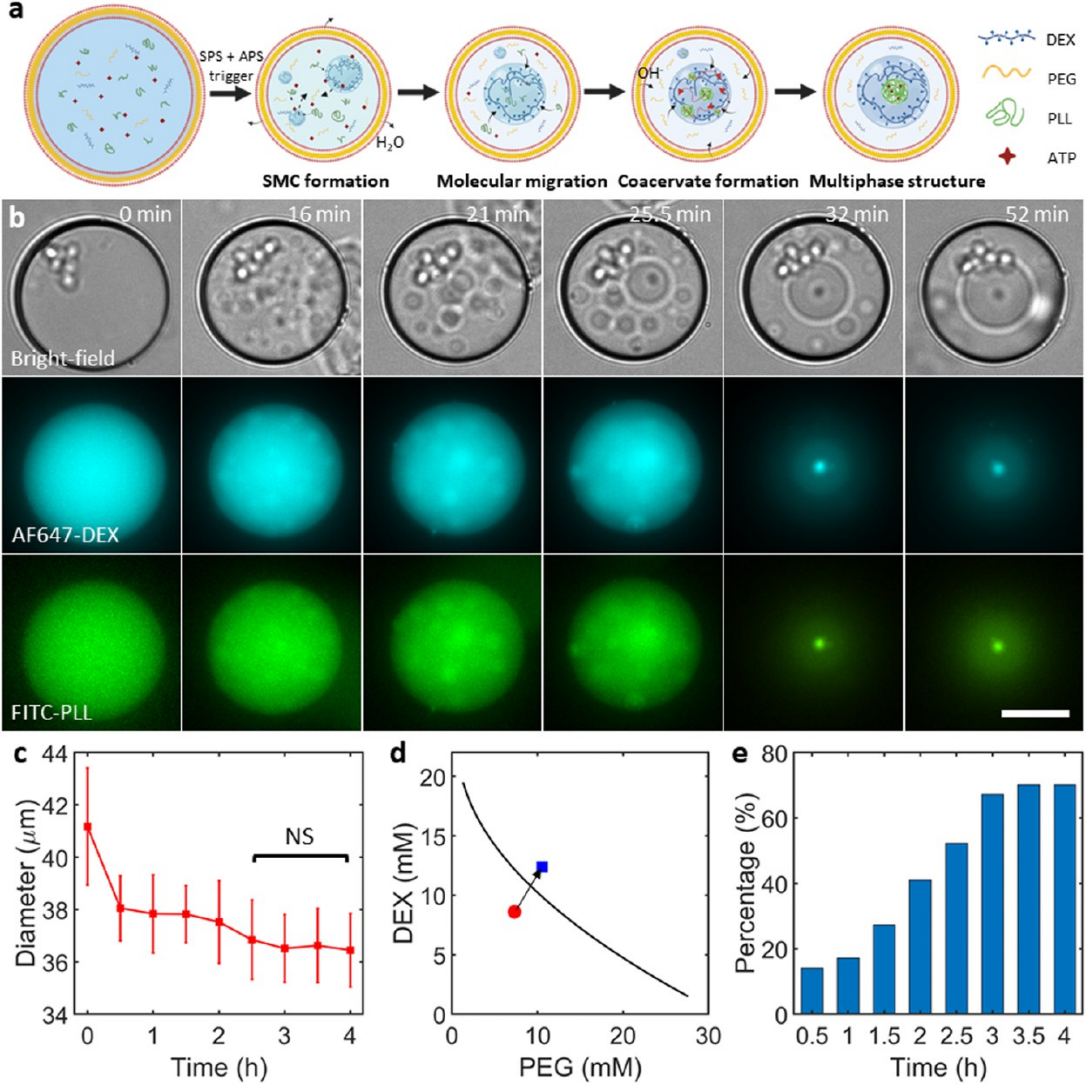
SMC-restricted coacervate dynamics within double emulsion compartments. (a) A conceptual sketch showing coacervate formation and recruitment by SMC, leading to a coacervate-in-SMC structure in double emulsions. (b) Bright-field and fluorescence time-lapse images taken by wide-field fluorescence microscopy showing the initial state, coacervate formation, and their restriction within SMCs after feeding the double emulsions with a hypertonic, high-pH buffer. The bright-field images show double emulsion shrinkage as a result of the hypertonic trigger. The DEX fluorescence (shown in cyan) shows the initiation of SPS, eventually leading to a multiphase structure, with the inner DEX-rich phase marked by higher intensity. The PLL fluorescence (shown in green) shows a process almost identical to that of the DEX channel, eventually forming a coacervate within the SMC. The images at 0 min show a representative double emulsion before the trigger. The last two fluorescence panels are taken from the bottom plane, while the rest are all captured at the equatorial plane. Scale bar, 20 μm. (c) Corresponding mean diameters of the double emulsion population showing a significant reduction in size for the first 2 h after the trigger; no significant difference (*p* > 0.05, marked NS) was observed after 2.5 h. Data points represent mean values while the error bars indicate standard deviations (*n* ≥ 55 double emulsions for each data point). (d) The initial concentrations of the encapsulated PEG and DEX (red circle) cross the binodal (black curve, obtained by fitting the experimental data) after the trigger (blue square, calculated from the volume change). (e) Plot showing the percentage of double emulsions containing coacervates in SMCs after the trigger (*n* ≥ 61 double emulsions for each data point). For all of the experiments, the oil phase was fluorinated oil (HFE 7500) containing 2% FluoSurf-C surfactant. The encapsulated solution consisted of 7.3 mM DEX, 8.6 mM PEG, 2.4 mg/mL PLL, 0.8 mM ATP, and 15% v/v glycerol in 15 mM citrate-HCl (pH 4). The double emulsion suspension was combined with an equal volume of feeding aqueous solution containing 500 mM sucrose and 15% (v/v) glycerol in 15 mM Tris-HCl (pH 9).

To further investigate the regulation of coacervates within a segregative system, we triggered APS and SPS simultaneously by mixing the four components under phase separation conditions; i.e., DEX and PEG concentrations were above the binodal, and the pH (6.3) was suitable for PLL/ATP conservation. Using wide-field fluorescence microscopy, we obtained multiphase emulsion droplets (Figure 1e), where PLL/ATP droplets were formed and confined within the DEX-rich SMCs, segregated from the PEG-rich environment. We also noted strong partitioning of DEX in the formed condensates, clear from the fluorescence overlap of Alexa Fluor 647 (AF647)-labeled DEX and fluorescein isothiocyanate (FITC)-labeled PLL. We observed the coalescence of SMC domains as well as of the coacervates present inside. As Figure 1f shows, PLL/ATP condensates readily fused with each other upon physical contact, followed by their relaxation into a bigger spherical condensate, indicating their liquid-like behavior within the SMC. However, physical contact was possible only for the coacervates within the same SMC domain. Figure 1g and Supporting Movie 1 show the coacervate movement strictly within the SMC domains, even when it is accentuated by an external fluid flow. Thus, SMC domains acted as incubation chambers for the condensates and physically restricted them.

Next, we used fluorescence recovery after photobleaching (FRAP) to examine the diffusion of coacervate components in such a setting by confocal laser scanning fluorescence microscopy (CLSM). To check this systematically, we bleached the FITC-PLL fluorescence of entire coacervates in four different environments and tracked their recovery (Figure 1h–i): (i) without any DEX or PEG; (ii) in the presence of 12 mM DEX; (iii) in the presence of 12 mM PEG; and (iv) in the presence of 12 mM PEG + 12 mM DEX (SPS conditions). In the first case, we obtained a fluorescence recovery of 85% within 20 min (Figure 1i—green curve). The addition of DEX reduced the extent of recovery to around 50% within the same time period (Figure 1i—red curve). In the presence of PEG, the recovery further decreased to only 22% (Figure 1i—black curve). This trend reached its highest extent for the coacervates in SMCs, where a meager 15% fluorescence was recovered after 20 min (Figure 1i—blue curve). The extent of recovery is affected by the PLL concentration in the surrounding dilute phase,^40^ especially since the entire coacervate was bleached. In the case where DEX and/or PEG was present, the decrease in recovery is likely aided by the higher viscosity of the surrounding environment, slowing the diffusion of PLL molecules. Additionally, the depletion effect by crowding agents has been demonstrated to induce the higher partitioning of PLL in the coacervate phase,^41^ further hindering the recovery. The lowest recovery in the case of coacervates confined within SMCs is a result of concentrated and thus highly viscous DEX-rich surroundings and confirms a chemically isolated environment, even for multiple coacervates present within the same SMC. We also observed concomitant differences in the diffusion coefficients of the PLL molecules (*D*_app,PLL_). Compared to the control case of no PEG or DEX (1.1 μm^2^/s), *D*_app,PLL_ in PEG and DEX solutions (both ≈0.6 μm^2^/s) was substantially lower (*p*-value ≤0.05, see Figure 1j), likely due to the increased viscosity.^42^ The coacervates confined within the SMC exhibited an even lower diffusion coefficient, *D*_app,PLL_ ≈0.4 μm^2^/s. Thus, these FRAP results indicate that the molecular diffusion between the coacervates and their environment, as well as with neighboring coacervates, is strictly restricted when the coacervates are confined within SMCs.

### SPS-APS Multiphase Structures within Nonmembranous Microconfinements

Based on the interactions between APS and SPS in the bulk, we next turned our attention to whether this hierarchical structure of membraneless compartments would still arise in confined synthetic cell models. We began by applying a simplified synthetic cell model: water-in-oil-in-water double emulsions (Figure 2a). The required APS and SPS components were efficiently encapsulated within double emulsions (osmolarity ≈70 mOsm) using a previously established microfluidic platform^24^ (see Materials and Methods for details). The oil phase consisted of a fluorinated oil (HFE 7500), containing 2% FluoSurf-C surfactant. The concentrations of the SPS components were kept below the binodal curve (Figure 1b) while the pH was kept at 4.5 in order to prevent coacervation. As the schematic (Figure 2a) and wide-field fluorescence microscopy images (Figure 2b) show, we simultaneously triggered both APS and SPS within the double emulsions using a hypertonic (500 mM sucrose) and high-pH (8.6) buffer. The hypertonic buffer caused water efflux, decreasing the double emulsion volume,^24^ and increasing the concentration of the encapsulated SPS components above the binodal,^23^ while the high-pH environment caused proton flux across the boundary, increasing the pH value of the lumen^24^ and inducing complex coacervation.^29^ Thus, post-trigger, the homogeneous interior of the double emulsions underwent both APS and SPS, resulting in multiphase structures. The cluster of bright spots seen in the bright-field images are small oil droplets formed during the production, were located outside of the double emulsions, and thus did not affect the phase separation processes under consideration. The process was similar to what was observed in bulk settings, in which DEX-rich phases readily fused with each other, eventually forming a single SMC, in which the coacervate was confined. Since the relatively thick oil shell (≈1 μm, see Supporting Figure 2) hindered the diffusion of water and hydroxyl ions, the construction of multiphase structures took several hours. We observed that while some double emulsions first showed SPS and others APS, all of them ultimately formed a similar multiphase structure. Similar to bulk experiments (Figure 1e), we observed increased AF647-DEX fluorescence in the coacervate, suggesting a secondary DEX enrichment within the coacervate.

Figure 2c shows the size variation of double emulsions post-trigger. The double emulsions shrunk from ≈41 to ≈38 μm in the first half an hour and then showed a gradual but still noticeable shrinkage over the next 3 h before reaching a plateau. Afterward, their size remained constant at ≈36.5 μm. Based on the observed volume reduction, concomitant increases in the encapsulated DEX and PEG concentrations were calculated (Figure 2d, see Materials and Methods for details). These final concentrations were above the binodal curve, aligning with the observed SMC formation. As shown in Figure 2e, the proportion of double emulsions containing multiphase structures increased rapidly until it plateaued at 70% after 3.5 h. We noted that the obtained multiphase structures were not stable over long-term as the coacervate gradually dissolved over 12 h (see Supporting Figure 3). Nonetheless, we were able to construct a multilevel compartment within double emulsions over the time scale of several hours, with the MSCs effectively restricting the coacervates.

### SMCs Recruit Coacervate Components to the Lipid Membrane

To further understand the role of the SPS regulation in biomimetic systems, we turned to liposomes, aqueous confinements surrounded by a lipid bilayer. We used octanol-assisted liposome assembly (OLA, see Supporting Figure 4),^43–45^ a liposome generation-visualization microfluidic technique, to conduct these experiments. The membrane composition was 99.9% 1,2-dioleoyl-*sn*-glycero-3-phosophocholine (DOPC) and 0.1% 1,2-dioleoyl-*sn*-glycero-3-phosphoethanolamine-*N*-(lissamine rhodamine B sulfonyl) (Rh-DOPE) (molar ratio); the latter was added for fluorescent visualization. Based on the well-known interaction between the DEX-rich domain and lipid membranes,^15,33,34^ we thought of directing the APS molecules to the membrane via SMCs. As shown in Figure 3a,b, cell-sized (10–20 μm in diameter) liposomes were produced, encapsulating the APS and SPS components in 25 mM Citrate-HCl buffer with pH 4.2. To start with, we solely triggered SPS by adding an equal volume of feeding aqueous solution (containing 600 mM sucrose in 25 mM Tris-HCl buffer, pH 9.0) to the liposome suspension (see Materials and Methods for details). The final pH of the mixture was measured to be 5.1 due to buffering; thus, no induction of APS is to be expected. We further demonstrated only SPS formation by triggering the same system in double emulsions, which eliminates any membrane interactions and thus makes it easier to assess the phase separation, and exclusively observed SPS formation (Supporting Figure 5). As Figure 3b shows, a clear shrinkage in the liposome size was observed after feeding (panels at 384 s), confirming the expected response to the hypertonic environment. Meanwhile, multiple SMCs formed, as evidenced by the increased DEX fluorescence. As expected, there was an overlap of PLL and DEX fluorescence within SMC regions, indicating the recruitment of PLL.

Notably, the SMCs, with PLL recruited inside, readily interacted with the phospholipid membrane. By scanning across different planes, all liposomes within a batch (*n* = 79) were observed to have formed bud-like structures. Figure 3c shows an example, displaying two planes of a liposome 30 min post-trigger. In the plane of the SMC bud, marked by a circular lipid structure on the membrane surface, high DEX and PLL fluorescence intensities were observed within the buds. The equatorial plane (inset) shows the nonbudded part of the liposome with no SMC localization at the membrane. An intensity profile across the bud (dotted line in Figure 3c), showed a strong peak in both DEX and PLL fluorescence, as well as increased lipid fluorescence (Figure 3d). This overlap of increased lipid fluorescence confirms the pronounced nature of these SMC buds while being covered by the lipid membrane.

Due to the recruitment within SMCs, PLL molecules were transported to the membrane surface. By plotting the PLL fluorescence intensity as a function of the distance from the center of the liposomes, the PLL fluorescence was indeed observed to be localized at the membrane (Figure 3e, *n* = 10). Furthermore, these PLL-enriched SMCs at the membrane could be seen undergoing fusion events (Figure 3f). As the two SMCs (indicated by white arrows in Figure 3f) came into contact, they merged into a single entity (indicated by green arrows in Figure 3f). These fusion events suggest that the coverage of SMCs by the membrane is incomplete, resulting in protruding buds that are open at the base. Apart from the fusion, the budded SMCs also showed two-dimensional diffusion on the membrane surface (Supporting Movie 2). Figure 3g shows the trajectories of several membrane-bound SMCs, restricted along the membrane surface. As seen in Figure 3h, their mean square displacement (MSD) increased linearly in time (*R* ^2^ = 0.99), indicating an unrestricted two-dimensional (2D) diffusion.^46^ Thus, even if the budded SMCs are coupled to the membrane, they, and as a result the sequestered cargo, are free to diffuse along the membrane.

In conclusion, we demonstrated the transport capability of SMCs for APS components toward the lipid membrane as well as their diffusive motion at the membrane surface. This targeted recruitment may facilitate triggering coacervation at the membrane, to which we then turned our attention.

### Regulation of Coacervates by Membrane-Interacting SMCs

Based on the SPS-induced PLL migration, we further explored the possibility of inducing coacervation at the membrane (Figure 4a). For this, we encapsulated the same components in liposomes with the same lipid composition as above but with a weaker buffering capacity (15 mM citrate-HCl, pH 4.5) and applied a similar hypertonic (500 mM sucrose) buffer (15 mM Tris-HCl) at pH 8.6. The resulting pH after mixing the solutions in equal parts was 6.3, which was sufficient for APS triggering. This was also demonstrated by triggering both SPS and APS in the double emulsions using the same encapsulated components and triggers (Supporting Figure 6). Figure 4b shows time-lapse images of the process in liposomes taken by wide-field fluorescence microscopy (also see Supporting Movie 3). As can be seen, both SPS and APS occurred within minutes inside the liposome, along with the expected shrinkage of the liposome. The SMCs initially appeared at the membrane or within the liposome interior, after which they all wetted the membrane surface and reshaped the liposome into a “flower-like” shape. The SMC domains, representing the “petals”, showed strong partitioning of PLL into them. Simultaneously, PLL/ATP coacervates emerged, predominantly near the membrane, and eventually remained confined within the SMCs and in close vicinity of the membrane. Noticeably, unlike in the case of double emulsions (Figure 2), SMCs with coacervates did not remain suspended in the lumen but ultimately migrated to the membrane surface.

Supported by CLSM, Figure 4c and Supporting Movie 4 show the 3D rendering of the morphology of a liposome, captured 20 min post-trigger. Multiple DEX-rich petals (in cyan) could be seen, restructuring the liposome into a flower shape. The liposomes showed different morphologies in different planes due to the random distribution of the petals, some of which harbored the formed coacervates (in green) (see Supporting Movie 5 and Supporting Information Figure 7). Fluorescence-based frequency maps for DEX and PLL further clarified the DEX-rich nature of the petals and the strong confinement experienced by the coacervates within them (Figure 4d, see Materials and Methods for details). Since the formed DEX-rich domains fuse with each other upon physical contact in a diffusion-limited manner without any control, it is challenging to control the exact number of SMCs formed. However, coarsening of SMCs is significantly slowed once in contact with the membrane. Focusing on the number of petals formed at the equatorial plane showed a range of 1 to 8 petals per liposome (*n* = 34 different liposomes), 20 min post-trigger. As Figure 4e shows, after 20 min of incubation in a high-pH, hypertonic environment, 52% of the liposomes (*n* = 27) maintained multiple “petals” as opposed to a single SMC; 57% of these SMCs contained coacervates. As demonstrated previously, the formed ‘petals’ can remain stable for several hours since the membrane segment between them is deformed and gives rise to a net interbud repulsive force, elevating the activation energy barrier for bud coalescence.^23^

Similar to the bulk results, the DEX fluorescence intensity heat map based on confocal images clearly showed sequential DEX recruitment in the flower-shaped liposomes (Figure 4f). The first recruitment happened in the petals, where the DEX intensity was 2.5 times that of the lumen (Figure 4g). Further fluorescence enhancement occurred at the condensates trapped within, showing about 1.7 times further increase in the intensity. As pointed out by the arrow in the confocal image (Figure 4h), the confined coacervates showed budding behavior, protruding away from the liposome but still surrounded by the lipid membrane. Based on these observations, we concluded that both SMCs and coacervates interact with membranes, forming relatively large petal-like and much smaller bud-like protrusions, respectively.

**Figure 3:**
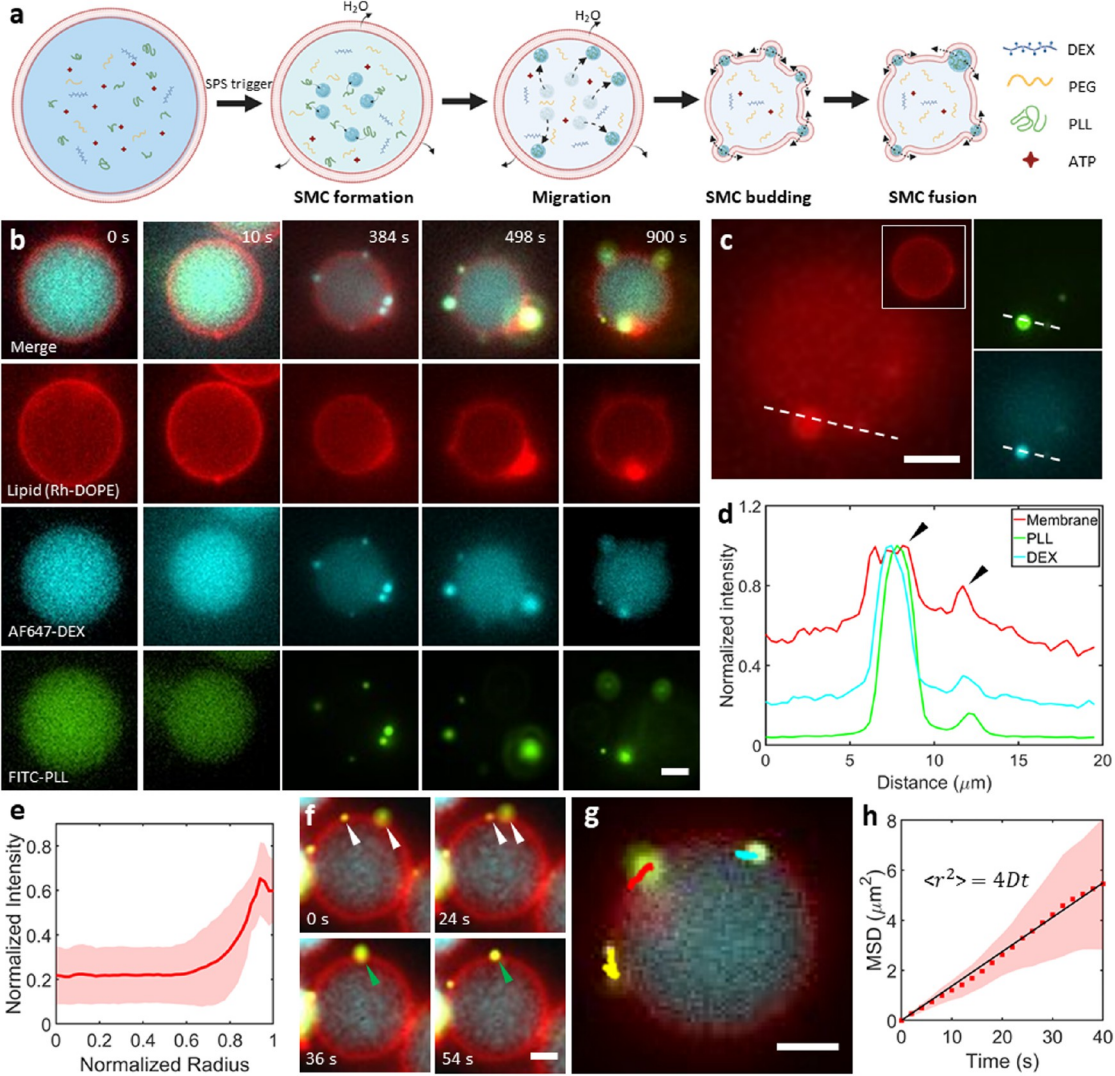
SMCs bud on the membrane and recruit APS components. (a) Conceptual sketch showing SMC formation and recruitment of PLL molecules, ultimately resulting in budded structures at the membrane that show a restricted 2D motion and are semienclosed by the membrane. (b) Fluorescence time-lapse images showing the initial state, followed by SMC formation, PLL recruitment, membrane localization, and budding upon trigger. The budded SMCs can be seen as intense bright dots (cyan) appearing at the membrane over time. The PLL channel (green) shows a strong sequestration of PLL in the SMCs. The panel at 0 s shows a representative liposome before the trigger. (c) Fluorescence image of the liposome surface showing the budded structures; the inset illustrates the equatorial plane, showing the overall spherical nature of the liposome. The images on the right show the PLL (top panel) and DEX (bottom panel) fluorescence in the bud. (d) Line graphs corresponding to the dotted lines in (c) show the colocalization of the lipid bud, SMC, and the sequestered PLL molecules. (e) Mean PLL fluorescence as a function of the normalized liposome radius (*n* = 10 different liposomes). In (c–e), the data were collected 30 min after the trigger. (f) Time-lapse showing a fusion event between two membrane-bound SMCs. (g) Trajectories of SMCs over a course of 22 s, showing their 2D-restricted movement on the membrane surface. (h) A plot showing a linear increase in the MSD of the budded coacervates. (*n* = 5 coacervates in 4 different liposomes). The black line shows a linear fit (*R* ^2^ = 0.99). In (b–g), the images were taken by wide-field fluorescence microscopy. In (f–h), the data were collected 20 min after the trigger. For all experiments, the lipid composition was 99.9% DOPC + 0.1% Rh-DOPE (molar ratio). The encapsulated solution consisted of 7.3 mM DEX, 8.6 mM PEG, 2.4 mg/mL PLL, 0.8 mM ATP, and 15% v/v glycerol in 25 mM citrate-HCl (pH 4). The liposome suspension was combined with an equal volume of feeding aqueous solution containing 600 mM sucrose and 15% (v/v) glycerol in 25 mM Tris-HCl (pH 9). All scale bar, 5 μm. Data points represent mean values, with shaded areas indicating standard deviations.

Importantly, the coacervates were restricted not only by SMCs, but also by their budding behavior. The individual trajectories in each petal shown in Figure 4i confirm the random but restricted motion of coacervates (see Supporting Movie 6). Due to the separation of the SMCs, the coacervates remained isolated and could not come in contact with others residing in different petals. We analyzed the diffusive behavior of the coacervates, based on their MSDs. While the MSD trajectory increased linearly at the beginning, it soon plateaued for longer time periods (Figure 4j), indicating this diffusion was free at short times but the effect of barriers became dominant at longer times. Such diffusive motion, exhibiting free movement but within a bounded region, resembled a corralled diffusion.^47^ Indeed, the corresponding equation,^46^ MSD ≃ ⟨*r*_c_^2^⟩[1 – *A*_1_ exp­(−4*A*_2_ *Dt*/⟨*r*_c_^2^⟩)], where ⟨*r*_c_^2^⟩ is the corral size, *D* is diffusion coefficient, and *A*_1_ and *A*_2_ are constants determined by the corral geometry, fitted the observed MSD behaviors very well (*R* ^2^ = 0.99). The corresponding average corral size was 0.7 μm^2^, representing the average restricted area provided by the SMCs. Comparing the short-range diffusion of these confined coacervates with that of the membrane-bound SMCs (Figure 3h) indicated that the diffusion coefficient of the SMC buds was 2.8 times higher than that of the coacervate buds (Figure 4k). This slower diffusion of the confined coacervate buds is likely caused by the higher viscosity and the crowded environment in DEX-rich SMCs.

Thus, we demonstrated APS-membrane interplay with SMCs acting as mediators between the membrane and the lumen. Such SPS-based regulation of coacervates can prove to be useful in directed compartmentalization within engineered synthetic cells.

**Figure 4:**
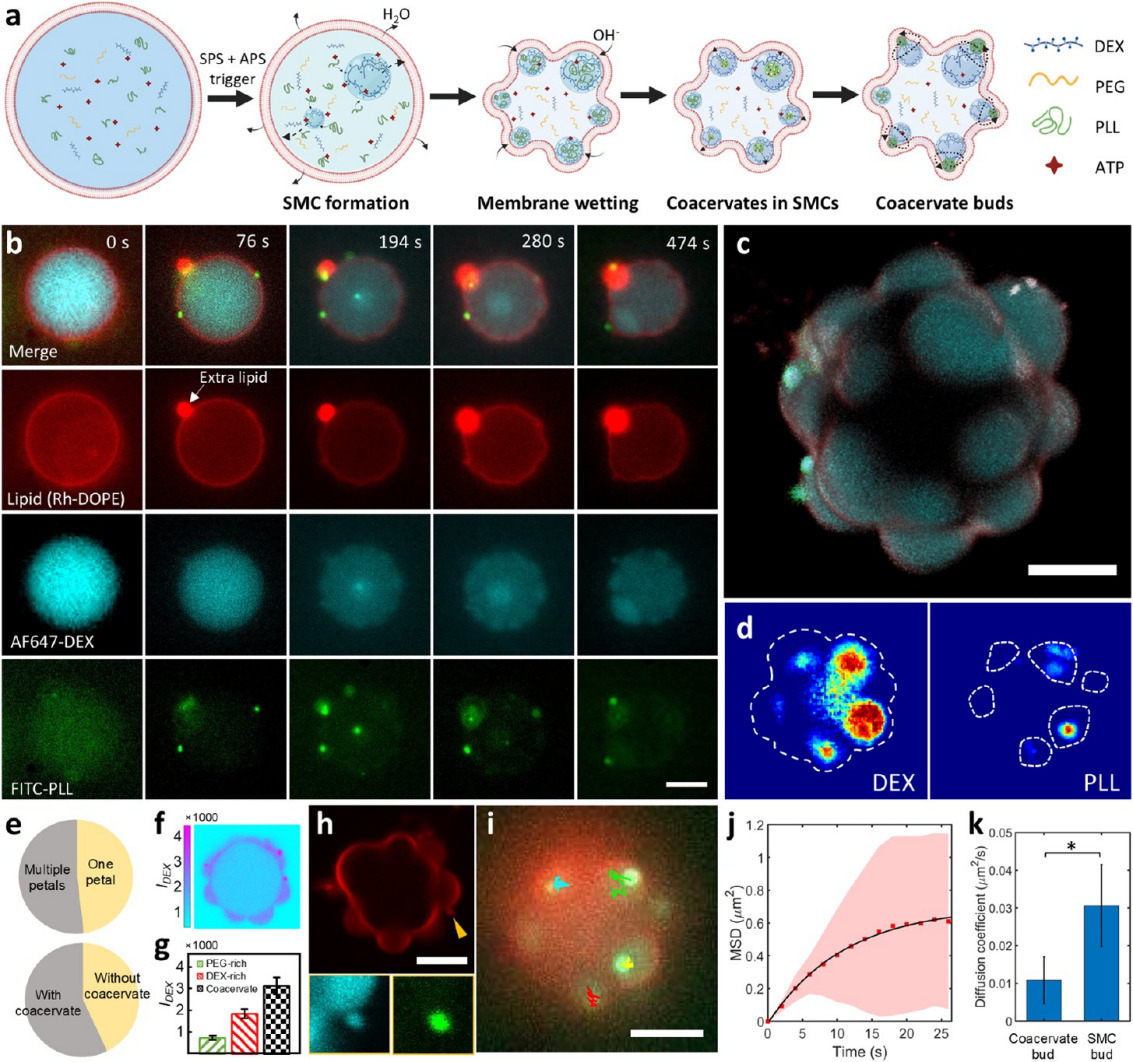
SPS domains regulate coacervates at the membrane. (a) Conceptual sketch showing the formation of SPS domains at the membrane, triggering of PLL/ATP condensation, and subsequent restricted coacervate motion within the SPS domains. (b) Fluorescence time-lapse images taken by wide-field fluorescence microscopy showing the above-mentioned stages after exposing the liposomes to a hypertonic, high-pH buffer. Volume reduction of the liposome is visible in the lipid channel (in red), sometimes with the extra lipids forming a pocket as indicated. Over time, DEX-rich domains (in cyan) were formed, underwent coalescence, and ultimately wetted the membrane. These domains recruited coacervates to the membrane (observed as intense green dots). (c) 3D projection based on CLSM images showing the final morphology of a liposome with petal-shaped SPS domains, some containing condensates. (d) Fluorescence-based frequency heat maps showing the observed occurrence frequency (cumulative over one min) for DEX-rich domains (left panel) and PLL/ATP condensates (right panel) in a liposome. The dotted lines are indicative of the lipid membrane (left panel) and DEX-rich domains (right panel). (e) Pie charts showing the percentage of liposomes with multiple petals (top panel) and the percentage of petals containing condensates (bottom panel); *n* = 27 different liposomes. (f) Fluorescence intensity heat map showing DEX distribution in a single plane of the liposome, constructed from a confocal image. (g) A bar chart showing dextran-partitioning in PEG-rich phase, DEX-rich phase, and the condensate region; *n* = 5 different liposomes. (h) A confocal image of the liposome, showing the budded coacervate region (pointed by the arrow) visualized in the lipid channel, while the zoom-ins show the same region in DEX (left panel) and PLL (right panel). (i) Trajectories of the coacervates recorded by wide-field fluorescence microscopy showing their restricted motion within the membrane-bound SMCs. (j) MSD plot showing the confined motion of condensates within membrane-bound SMCs; *n* = 4 coacervates within the same liposome. The black curve shows the corralled diffusion fit (*R* ^2^ = 0.99). (k) Comparison of the diffusion coefficients between the coacervate buds and the SMC buds (from the previous section); *n* ≥ 4 coacervates; *p*-value <0.05. In (c–k), the data were collected 20 min after the trigger. For all experiments, the lipid composition was 99.9% DOPC + 0.1% Rh-DOPE (molar ratio). The encapsulated solution consisted of 7.3 mM DEX, 8.6 mM PEG, 2.4 mg/mL PLL, 0.8 mM ATP, and 15% v/v glycerol in 15 mM citrate-HCl (pH 4). The liposome suspension was combined with an equal volume of feeding aqueous solution containing 500 mM sucrose and 15% v/v glycerol in 15 mM Tris-HCl (pH 9). All scale bar, 5 μm.

## Conclusions

It is well-established that preferential interactions between constituent molecules can destroy homogeneity, drive demixing, and produce coexisting phases within cellular systems. But how these interactions, occurring simultaneously and dynamically within the cytoplasmic space, influence each other is still largely unknown. Using a model system consisting of four components in synthetic giant vesicles, we have demonstrated that SPS can regulate APS at both the molecular and coacervate level. We further showcase that the APS–SPS–membrane interplay can generate an entirely different degree of freedom, regulating dynamic reorganization, spatial localization, movement restriction, and surface interactions of condensates within the crowded aqueous space.

We show molecular enrichment via SPS-induced membrane-free confinements (SMCs), recruiting and transporting molecules to the membrane (or keeping them in the lumen), allowing further condensation under the right conditions. Since synthetic biomolecular condensates have been well demonstrated to mimic^48,49^ as well as enhance cellular functions,^50^ such membrane-targeted migration of molecules may further improve the efficiency of their on-membrane or transmembrane reactions. Thus, it will be beneficial to assess the generality of such affinity-based compartmentalization in diverse chemical systems including elastin-like^51–53^ and other polypeptides,^39,54^ RNA,^55^ or even nanoliposomes.^56^ Interestingly, SMCs have also been demonstrated to partition nonbiorelevant components like metal particles^57^ or organic polymers.^58^ These substances, which normally do not exist in living organisms, may bring new functionalities to synthetic cells.^59^

We further demonstrated that SMCs can spatially restrict coacervate movement. Moreover, due to the lack of molecular communication across coacervates,^26^ even within the same SMC, each coacervate can be considered independent. Therefore, for reaction cascades, these coacervate-in-SMC multiphases may be a good strategy for providing a membrane-free domain. Also, as the observed SPS is mainly driven by concentration and is stable across a wider pH range, one can easily switch the electrostatic environment to tune APS individually.^60^ Such a hierarchical multiphase assembly with distinct triggers allows for more flexible and dynamic control.

While high-throughput single emulsion microfluidics^61,62^ provides an efficient solution for studying subcompartmentalization,^63^ the phase separation largely depends on the encapsulated components rather than the subsequent external stimuli,^64^ making the single emulsion system relatively inflexible. The double emulsion system we used here provides the ability to trigger processes within the containers simply by tuning the extracellular environment.^24^ Notably, double emulsions are robust in various physicochemical variations including pH, temperature and osmotic pressure,^24^ making them a handy system if membrane interactions^65,66^ are not necessary or to be deliberately avoided.^24^ To quicken the response of the encapsulated contents to external stimuli, reducing the thickness of the oil shell during the production process or adjusting the oil composition could be effective strategies.

When it comes to the use of liposomes, APS–membrane interactions are facilitated via the SMCs. It is worth noting that the DOPC we used here should confer a slight negative charge on the membrane.^67^ Meanwhile, dextran molecules also exhibit a moderately negative zeta potential.^68^ Thus, the membrane interactions that we observe are not charge-driven but rather a result of the energetically favorable wetting of the membrane by the DEX-rich SMCs.^33,69^ The observed charge-independent membrane attachment here could be applied to simplify synthetic cells when studying coacervate-membrane interactions. Additionally, more often than not, APS–membrane interactions are studied from the outer leaflet, partly owing to the difficulty of encapsulation.^28,31,32^ Because of our microfluidic approach,^43–45^ we are able to study them from inside the liposomes, allowing us to mimic a more natural system.

In synthetic cell confinements, the isolation of coacervates as individual reaction hubs is highly significant but still requires exploration. Here, the membrane-bound SMCs offer numerous microchambers for the coexistence of multiple identical condensates within a single liposome. As SMCs can be stable for hours without fusion, they can provide independent compartments for their subordinate coacervates, and this stability could be further prolonged using lipids with different fluidities.^23^

Taken together, our results highlight a heretofore unappreciated factor, segregative phase separation, which produces distinct functional levels of condensate regulation in synthetic cells. Our findings suggest a universal physicochemical principle and a nonspecific biological strategy to spatially and temporally regulate biomolecular condensates. This design principle has broad applications in controlling the behavior of membraneless organelles and constructing diverse synthetic cell architectures.

## Materials and Methods

### Materials

Dextran (MW 9–11 kDa, No. D9260), poly­(ethylene glycol) (MW 8 kDa, No. 89510), poly-l-lysine (MW 15–30 kDa, P7890), poly-l-lysine–FITC-labeled (MW 15–30 kDa, P3543), adenosine-5′-triphosphate disodium salt hydrate (ATP, A2383), sucrose (S0389), sodium citrate tribasic dihydrate (citrate-base, 71405), tris­(hydroxymethyl)­aminomethane (Tris-base, 252859), poly­(vinyl alcohol) (PVA, average MW 30–70 kDa, P8136), 1-octanol (297887), glycerol (G2025), Pluronic F68 nonionic surfactant (24040032), ECO Tween-20 (STS0200), and hydrochloric acid were purchased from Sigma-Aldrich. Labeled dextran (Alexa Fluor 647, 10,000 MW, D22914) was purchased from Fischer Scientific B.V. Phospholipids, DOPC (SKU: 850375C) and Rh-DOPE (SKU: 810150C) were purchased from Avanti Polar Lipids, Inc. *N* ^6^-(6-Aminohexyl)-adenosine-5′-triphosphate labeled with cy3 (NU-805-CY3) was purchased from Jena Bioscience. Sylgard 184 silicone elastomer (PDMS) and curing agent were purchased from Dow. Silicon wafers were bought from Silicon Materials. Photoresist (EpoCore 10) and photoresist developer (mr-Dev 600) were purchased from Micro Resist Technology GmbH. Microfluidic accessories including a liquid flows tygon tubing coil 1/16″ OD X 0.02″ ID (SKU: LVF-KTU-13), stainless steel 90° Bent PDMS couplers (SKU: PN-BEN-23G), rapid-core microfluidic punches (*D* = 0.5 and 3 mm), PTFE Tubing 1/16″ OD (SKU: BL-PTFE-1608–50), and HFE 7500 fluorinated oil containing 2% FluoSurf-C surfactant (SKU: EU-FSC-V10–2%-HFE 7500) were purchased from Darwin Microfluidics. Elveflow pressure controller OB1-MK3 was used to control the fluid flow.

### Stock Solutions

Stock solutions of DEX (30 mM), PEG (70 mM), and sucrose (1 M) were prepared by dissolving the respective materials in Milli-Q water using a volumetric flask. On average, 40 mM DEX 10k is equivalent to ≈34 wt % while 70 mM PEG 8k is equivalent to ≈51 wt %.

The lipid stock solutions were prepared as described in detail previously.^45^ In this paper, we used a mixture of DOPC and Rh-DOPE (molar ratio = 1000:1). Briefly, we pipetted an appropriate volume of DOPC and Rh-DOPE chloroform solutions at the bottom of the round-bottom flask. The chloroform was fully evaporated by passing a gentle stream of nitrogen into the flask and desiccating the flask for more than 2 h to form a dry lipid film at the bottom. A 10% w/v lipid stock was then made by dissolving the lipids in an appropriate volume of ethanol. For long-term preservation, the stock was stored in a dark glass vial filled with an inert atmosphere at −20 °C.

### Elucidation of the Phase Diagram

The binodals of PEG and DEX were measured at room temperature using a variation of cloud-point titration.^70^ A known volume of a DEX stock solution was added to a small glass vial. A small known volume of PEG stock solution was then added, and the mixture was stirred. This process was repeated until the resulting mixture reached its cloud point and became turbid. Milli-Q water was then added in small volume increments to the mixture, followed by mixing until the mixture became clear again. Alternating volumes of the PEG stock and milli-Q water were titrated into the mixture, crossing above and below the cloud-point, until the cloud-point could not be reached or a significantly large volume of PEG was required. The process was then repeated switching DEX and PEG. Subsequent binodals that include different buffers or glycerol were produced by adding equal concentrations to each of the stock solutions and water, so the concentration of the added component would remain constant. The procedure was otherwise identical.

### Measurement of Partition Coefficients

The partitioning of PLL or ATP in a phase-separated DEX/PEG mixture was measured by mixing the corresponding components in Eppendorf tubes. Each mixture consisted of PEG and DEX in varying concentrations in 25 mM Tris-HCl at pH 7.4 and with either 2.4 mg/mL PLL (unlabeled: FITC-labeled = 10:1, mass ratio) or 0.8 mM ATP (unlabeled: cy3-labeled = 1000:1, molar ratio). The concentration of PEG was varied between 20, 15, and 10 wt % while DEX varied between 15, 10, and 5 wt %, resulting in nine total combinations of PEG and DEX. These vials were vortexed to ensure mixing and then left in the fridge overnight to allow the PEG and DEX to phase separate. On a clean glass slide, 5 μL droplets were placed from either the top PEG-rich phase or the bottom DEX-rich phase and immediately imaged using fluorescence microscopy. The average fluorescence intensity was normalized as follows^71^

$$I=I_{\mathrm{measured}}-I_{\mathrm{reference}}$$

where *I* is the normalized intensity, *I*_measured_ is the measured intensity in droplet and *I*_reference_ is the mean intensity in a random area outside the droplet. The partitioning coefficients of the fluorescent components, *K*_PLL_ or *K*_ATP_, were calculated by

$$K=\frac{I_{\mathrm{DEX}}}{I_{\mathrm{PEG}}}$$

where *I*_DEX_ and *I*_PEG_ are the normalized intensity of PLL/ATP in the DEX-rich phase and the PEG-rich phase, respectively. Each data point had three replicates.

### Microfabrication and Surface Functionalization

Master wafers were prepared according to the previously described microfabrication method^45^ and the channel heights were kept at 10 μm for OLA or 20 μm for double emulsion production. Microfluidic devices were prepared by the standard soft lithography method.^72–74^ Briefly, PDMS and the curing agent were mixed in a 10:1 weight ratio and then poured onto the master wafer and degassed using a vacuum desiccator. Meanwhile, a PDMS-coated glass coverslip (Corning no. 1) was coated with PDMS by spin coating at 500 rpm for 15 s (at an increment of 100 rpm/s) and then at 1000 rpm for 30 s (at an increment of 500 rpm/s). Both PDMS-covered wafer and PDMS-coated glass were baked at 70 °C for 2 h. The hardened PDMS block was carefully removed from the wafer; then, the inlet and outlet holes were punched using a biopsy punch of diameter 0.5 and 3 mm for the OLA (0.75 mm for double emulsion production). The PDMS block was then bonded on the PDMS-coated coverslip after 30 s of plasma treatment at 12 MHz (RF mode high) using a plasma cleaner (Harrick Plasma PDC-32G). The bonded device was then baked at 70 °C for 2 h.

After baking, the production chips underwent a PVA (5% w/v, molecular weight 30–70 kDa) treatment as described previously.^45^ Briefly, the outer aqueous inlet was flowed with a PVA solution, whereas the inner aqueous and oil inlets were kept at a positive pressure to retain the PVA-air boundary stable at the production junction. After 15 min of incubation, PVA solution was pushed out by applying maximum pressure (2 bar) on the inner aqueous and oil inlets and the extra liquid was removed by applying a negative pressure (−1 bar) at the exit. The device was then dried for 15 min by baking on a hot plate at 120 °C.

PDMS wells for FRAP and double emulsion experiments were prepared by using a clean silicon wafer without any pattern. First, nonpatterned PDMS blocks were prepared similarly to the description above by pouring a PDMS-curing mixture over the wafer, followed by baking and removing the cured material from the wafer. The blocks were then punched for 5 mm holes as the wells. After plasma bonding on a PDMS-coated glass slide, the wells were surface-functionalized by pipetting PVA solution (5% w/v) followed by 15-min incubation. The PVA was then pipetted out, and the device was baked on a hot plate at 120 °C for 15 min. After surface functionalization, all devices were stored at room temperature and stable for months.

### Bulk Experiments

In the bulk SPS–APS interaction experiment (Figure 1e–g), we made a mixture of 3 mg/mL PLL, 4 mM ATP, 12 mM DEX, 12 mM PEG and 15% glycerol in a buffer made by one-to-one mixing of 15 mM Tris-HCl (pH 9) and 15 mM citrate-HCl (pH 4) in an Eppendorf tube. After proper mixing by pipetting, 5 μL of solution was dropped on a cover glass and observed under the wide-field fluorescence microscope. A transparent lid was used to prevent evaporation.

For FRAP experiments (Figure 1h–j), we made four mixtures containing 3.6 mg/mL PLL, 4.8 mM ATP and 15% glycerol in a buffer made by one-to-one mixing of 15 mM Tris-HCl (pH 9) and 15 mM citrate-HCl (pH 4) in Eppendorf tubes: (1) no crowding agent, (2) 12 mM DEX, (3) 12 mM PEG, (4) 12 mM DEX, and 12 mM PEG. After proper mixing by pipetting, 10 μL of solution was dropped on a PDMS well with cover glass and observed under a confocal fluorescence microscope. For bleaching, the regions of interest (ROI) were entire condensates of approximately 14 μm in diameter, and they were bleached using 100% laser intensity for 100 frames with a frame interval of 51 ms. Intensity of the bleached area was normalized using the same method as described previously^75^

$$f\left(t\right)=\frac{I_{\mathrm{correct}}\left(t\right)-\min\left(I_{\mathrm{correct}}\right)}{I_{\mathrm{correct}}\left(0\right)-\min\left(I_{\mathrm{correct}}\right)}$$

where

$$I_{\mathrm{correct}}=C\left(t\right)\times I\left(t\right)$$

and

$$C\left(t\right)=\frac{R\left(0\right)}{R\left(t\right)}$$

*R*(*t*) and *I*(*t*) indicate the fluorescence intensity of the reference droplet at time *t* and the original fluorescence intensity of the bleached region at time *t*, respectively; min­(*I*_correct_) indicates the minimum value of *I*_correct_, which is obtained right after the sample is bleached. The normalized intensity was fitted using the following function

$$f\left(t\right)=A\left(1-e^{\left(-t/\tau \right)}\right)$$

where *A* and τ indicate the amplitude of recovery and the relaxation time, respectively. The apparent diffusion coefficient (*D*_app_) was calculated using the formula

$$D_{\mathrm{app}}≃\frac{\omega^{2}}{t_{\left(1/2\right)}}$$

where *t*_(1/2)_ is the half-life fluorescence recovery and ω^2^ is the area of the bleached cross section. The half-life *t*_(1/2)_ was calculated using the following formula

$$t_{\left(1/2\right)}=\ln\left(2\right)\tau$$

### Liposome and Double Emulsion Production

Octanol-assisted liposome assembly (OLA) method^43^ was used for liposome production. Four solutions were prepared: inner aqueous (IA), outer aqueous (OA), lipids in 1-octanol (LO), and exit well aqueous (EA). IA, OA, and EA always contained 15% v/v glycerol and a pH-regulating critrate-HCl buffer (pH ≈ 4). Additionally, 5% w/v F68 surfactant was always present in OA. Phase separation components coexisted in IA as a homogeneous solution: 7.3 mM DEX (unlabeled: AF647-labeled = 1000:1, molar ratio), 8.6 mM PEG, 2.4 mg/mL PLL (unlabeled: FITC-labeled = 5:1, mass ratio), and 0.8 mM ATP. The osmolarity of the aqueous solutions was balanced by the addition of 70 mM sucrose in OA and EA. The lipid-carrying organic phase was prepared by mixing 10% w/v lipid stock (i.e., 100 mg/mL DOPC mixed with Rh-DOPE in the molar ratio of 1000:1) with 1-octanol to a final concentration of 0.2% w/v. A detailed protocol is described elsewhere.^45^ After the three inlet pressures were adjusted and stable production was obtained, the open well was filled with 10 μL of EA to collect the liposomes. The production phase normally lasted for about 30 min.

Double emulsion production was conducted by a double-junction microfluidic design as described previously.^24^ We used similar IA and OA components as the liposome experiments except that the surfactant in OA was replaced by 1% v/v Tween-20. The oil phase was prepared by mixing labeled-lipid stock (100 μg/mL Rh-DOPE in ethanol) with HFE 7500 fluorinated oil (containing 2% FluoSurf-C surfactant) to a final Rh-DOPE concentration of 2 μg/mL. A clean pipette tip (200 μL) was inserted into the outlet to collect the produced double emulsions. The dispersion was stored in a dark glass vial and kept at 4 °C.

### On-Chip Treatment and Observation

In the liposome experiments, triggering was conducted on the same chip as the production chip. Briefly, phase separation was triggered by gently removing 5 μL of the solution so as to not disturb the settled liposomes and refilling the well with 5 μL of appropriate feeding aqueous (FA). The well was covered with a coverslip to prevent evaporation, except during solution exchange.

In the double emulsion experiments, triggering was performed by replacing the external solution, in which the double emulsions were dispersed. Initially, the PDMS well was filled by pipetting 90 μL of OA and 10 μL of double emulsion suspension. After the double emulsions sank to the bottom, 50 μL of the solution was removed from the top, followed by immediately refilling with 50 μL of FA. The well was consistently covered with a coverslip, except during solution exchange.

A detailed list of IA, OA, EA, and FA solution compositions for various experiments can be found in Supporting Table 1.

### Binodal Crossing Analysis

The binodal curve was fitted based on all experimental data in varied conditions (pH, viscosity, and ionic strengths) using the following equation

$$c_{\mathrm{DEX}}=A+B\times c_{\mathrm{PEG}}+C\times c_{\mathrm{PEG}}^{2}$$

where *A*, *B*, and *C* are the fitting constants, *c*_DEX_ and *c*_PEG_ are the concentrations of DEX and PEG respectively. Based on the fitted binodal curve, we further calculated the DEX and PEG concentrations after triggering. First, the volume ratio *R* was obtained as

$$R={\left(\frac{D_{\mathrm{before}}}{D_{\mathrm{after}}}\right)}^{3}$$

where *D*_before_ and *D*_after_ are the average diameters of the double emulsions before hypertonic high-pH feeding and 4 h postfeeding, respectively. The DEX and PEG concentrations inside the liposomes were then calculated as

$$c_{\mathrm{after}}=c_{\mathrm{before}}\times R$$

where *c*_before_ and *c*_after_ are the concentrations of DEX and PEG before and after the phase separation trigger, respectively.

### Microscopy

Images for bulk and on-chip experiments were acquired using a Nikon-Ti2-Eclipse inverted fluorescence microscope equipped with pE-300ultra illumination system, Nikon Plan F 10× (numerical aperture, NA 0.3) objective, Nikon Plan Fluor 40× (NA 1.30) oil objective, or Nikon Plan Apo 100× (NA 1.45) oil objective, and appropriate filter sets (Semrock). In the case of fluorescence visualization, samples were excited using 2–10% light intensity and an exposure time of 10–100 ms. All images were acquired using a Prime BSI Express sCMOS camera.

FRAP experiments were performed on a Leica SP8-SMD microscope using a 63× (NA 1.2) water objective. For bleaching, the ROI was bleached using 100% laser intensity of a tunable white light laser source (LEICA TCS SP5 X). The recovery of the bleached area was recorded for approximately 20 min.

Confocal images were acquired using a Nikon C2 Confocal laser scanning microscope equipped with a Ti2 Illuminator-DIA system, Nikon Plan Apo 60× (NA 1.4) oil objective, and appropriate filter sets (Semrock).

### Image Analysis

ImageJ was used for image processing and analysis in the case of FRAP experiments, fluorescence intensity analysis, and size measurements. In the case of double emulsion experiments, only those double emulsions that were nonclustered and in focus were taken into account for the analysis. Error bars in the graphs indicate the standard deviation of the mean for the respective samples.

MATLAB R2019b was used for fluorescence analysis and fitting. For PLL distribution along the radius (Figure 3e), the membrane boundary was selected based on the lipid channel, and then the center of the liposome was calculated. Subsequently, the PLL fluorescence intensity from the center to the boundary was measured, normalized by the maximum intensity value, and plotted against the normalized liposome radius. In case of DEX/PLL fluorescence appearance frequency heat map (Figure 4d), pixels with an intensity higher than 150 were considered as an appearance of MSCs/coacervates in that frame. All frames in the video (1 min duration) were analyzed, and the cumulative number of occurrences in each pixel was summarized and expressed as the frequency heat map. For DEX fluorescence distribution (Figure 4f), the fluorescence intensity within the liposome for a single time frame was expressed as a heat map. To obtain the diffusion coefficient in case of coralled diffusion (Figure 4k), linear part of the data (first 10 seconds) was fitted.

## Supplementary Material



















## Data Availability

Data supporting the findings of this study are available within the paper, its Supporting Information, and Source Data. Any additional supporting data is available from the corresponding authors upon reasonable request.

## Notes

Compositions used for microfluidic experiments; binodal curves for PEG and DEX; frequency histogram of the oil shell thickness; images of DEs with/without coacervates in SMC domains under different conditions; liposome production using OLA; confocal fluorescence images of a “flower-shaped” liposome in different planes (PDF)
Restricted motion of coacervates within MSCs, accentuated by an external fluid flow (AVI)
Two-dimensionally restricted movement of PLL-recruited SMCs on the membrane surface (AVI)
Formation of SPS and APS after exposing the liposome to a hypertonic, high-pH buffer (AVI)
3D projection showing the morphology of a liposome 20 min after the APS and SPS were triggered (AVI)
Confocal fluorescence z-stack showing a liposome at different planes after the formation of APS in SMCs (AVI)
Corralled diffusion of coacervates in SMCs at the liposome membrane (AVI)
MATLAB codes used for image processing (Source Code) (ZIP)
Raw data associated with the figures (Source Data) (XLSX)

## Notes

**Statistics and Reproducibility** Experiments related to the binodal curve (Figure 1b and Supporting Figure 1) were single repeats for each condition. Experiments related to PLL/ATP partitioning (Figure 1c,d), APS–SPS dynamics (Figure 1e–g, with varied ATP concentrations), FRAP experiments (Figure 1h–j), double emulsions (Figure 2 and Supporting Figures 2, 3 and 6), and liposomes (Figure 3 with varied FA osmotic pressure strengths between 300 and 600 mOsm, Figure 4, and Supporting Figure 4 and 7) were repeated at least three times with similar results. The experiment of SPS triggering in double emulsions (Supporting Figure 5) was a single repeat.
