# Supplementary material for: Regulating Biocondensates within Synthetic Cells via Segregative Phase Separation
Source: ACS Nano. 2025 Apr 28;19(22):20550–63. doi: 10.1021/acsnano.4c18971 (PMC12164530; doi:10.1021/acsnano.4c18971)
Supplement: Supplementary file 1 [file nn4c18971_si_001.pdf]

Supporting information for Regulating biocondensates within synthetic cells via segregative phase separation

Chang Chen,[†] Caroline M. Love,[‡] Christopher F. Carnahan,[¶] Ketan A. Ganar,[†] Atul N. Parikh,[‡,¶,§,‖,⊥] and Siddharth Deshpande[*,†]

†*Laboratory of Physical Chemistry and Soft Matter, Wageningen University & Research, 6708 WE, Wageningen, The Netherlands*

‡*Department of Materials Science and Engineering, University of California, Davis, CA 95616, Davis, USA*

¶*Biophysics Graduate Group, University of California, Davis, CA 95616, Davis, USA*

§*Department of Biomedical Engineering, University of California, Davis, CA 95616, Davis, USA*

‖*Singapore Centre for Environmental Life Sciences Engineering, Nanyang Technological University, 636921, Singapore*

⊥*Institute for Digital Molecular Analytics and Science, Nanyang Technological University, 637551, Singapore*

E-mail: siddharth.deshpande@wur.nl

Supporting Table

Supporting Table 1: **Detailed overview of the compositions used for the inner aqueous (IA), outer aqueous (OA), exit well solution (EX) and feeding aqueous (FA).** Table values denote concentrations of the various constituents used in the experiments. Concentrations are denoted as (i/ii/iii/iv), where the numbers i-iv respectively indicate the concentrations used in i: Fig. 2 and Supporting Fig. 3, 6; ii: Fig. 3; iii: Fig. 4 and Supporting Fig. 7; iv: Supporting Fig. 5.

Compound	IA	OA	EA	FA
DEX (mM)	7.3/7.3/7.3/7.3			
AF647-DEX (μM)	7.3/7.3/7.3/7.3			
PEG 8k (mM)	8.6/8.6/8.6/8.6			
PLL (mg/ml)	2/2/2/2			
FITC-PLL (mg/ml)	0.4/0.4/0.4/0.4			
ATP (mM)	0.8/0.8/0.8/0.8			
Glycerol (%, v/v)	15/15/15/15	15/15/15/15	15/15/15/15	15/15/15/15
F68 (%, w/v)		0/5/5/0		
Tween-20 (%, v/v)		1/0/0/1		
Sucrose (mM)		70/70/70/70	70/70/70/70	500/600/500/600
Citrate-HCl, pH 4.50 (mM)	15/0/15/0	15/0/15/0	15/0/15/0	0/0/0/0
Citrate-HCl, pH 4.17 (mM)	0/25/0/25	0/25/0/25	0/25/0/25	
Tris-HCl, pH 8.64 (mM)				15/0/15/0
Tris-HCl, pH 8.95 (mM)				0/25/0/25

Supporting Figures

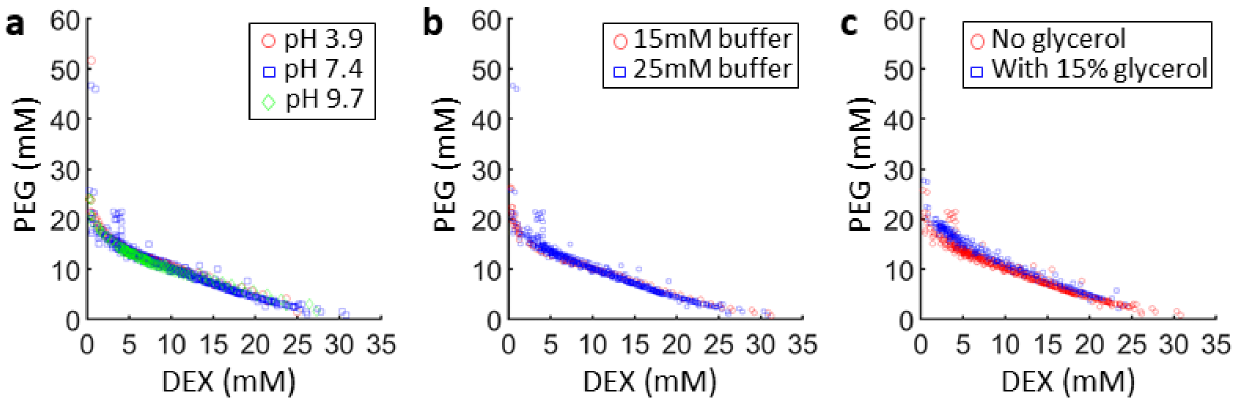

Supporting Figure 1: **Binodal curves for PEG (8 kDa) and DEX (10 kDa) at room temperature for three different parameter variations.** The binodal line was obtained by the cloud-point method in bulk solution in varied (a) pH, (b) buffer concentrations, and (c) viscosity.

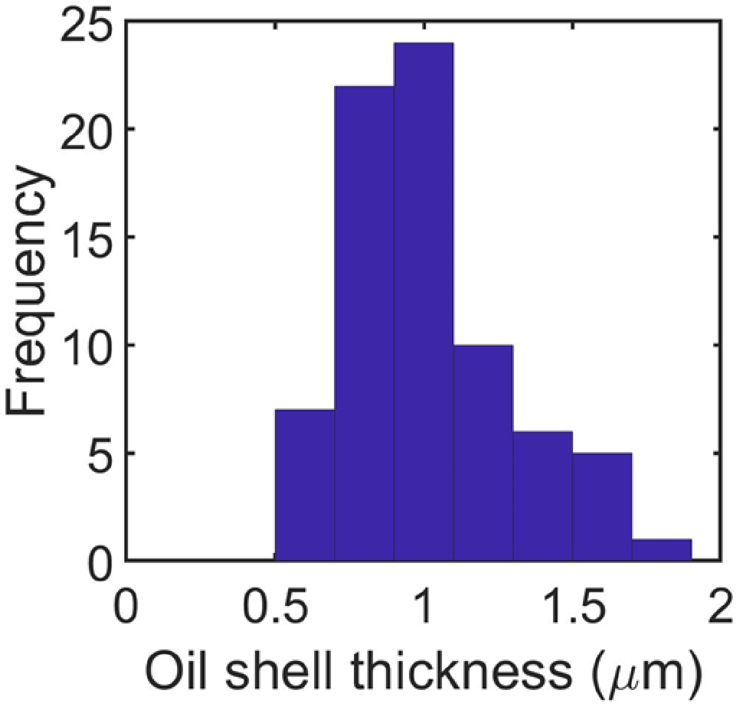

Supporting Figure 2: **Thickness of double emulsions.** Frequency histogram showing the thickness distribution of the oil shells that form the boundary of the double emulsions (n = 75 from a single production batch).

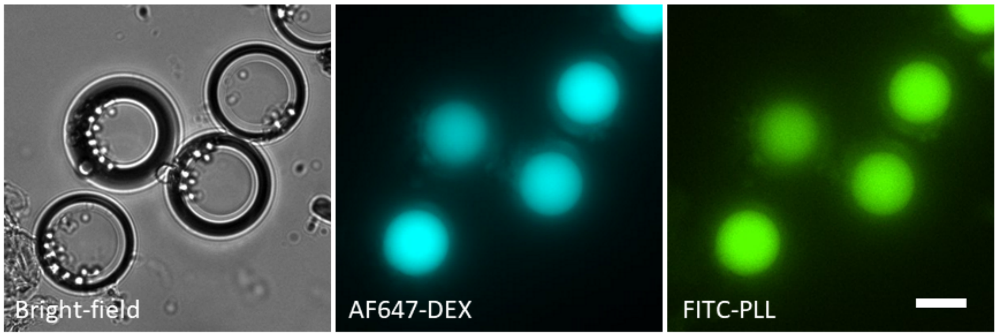

Supporting Figure 3: **Coacervates confined within SMCs, formed within double emulsions, tend to dissolve over longer duration.** Bright-field and corresponding fluorescence images, captured 13 hours after the hypertonic, high-pH trigger. The bright-field image shows a single SMC domain in each double emulsion, as confirmed by the DEX fluorescence. The PLL fluorescence shows complete overlap with DEX fluorescence, but no higher-fluorescence regions are present witihn the SMC domain, indicating coacervation dissolution. The IA solution consisted of 7.3 mM DEX, 8.6 mM PEG, 2.4 mg/ml PLL, 0.8 mM ATP, and 15% v/v glycerol in 15 mM citrate-HCl (pH 4). The double emulsion suspension was combined with an equal volume of feeding aqueous solution containing 500 mM sucrose and 15% v/v glycerol in 15 mM Tris-HCl (pH 9). Scale bar, 20 µm.

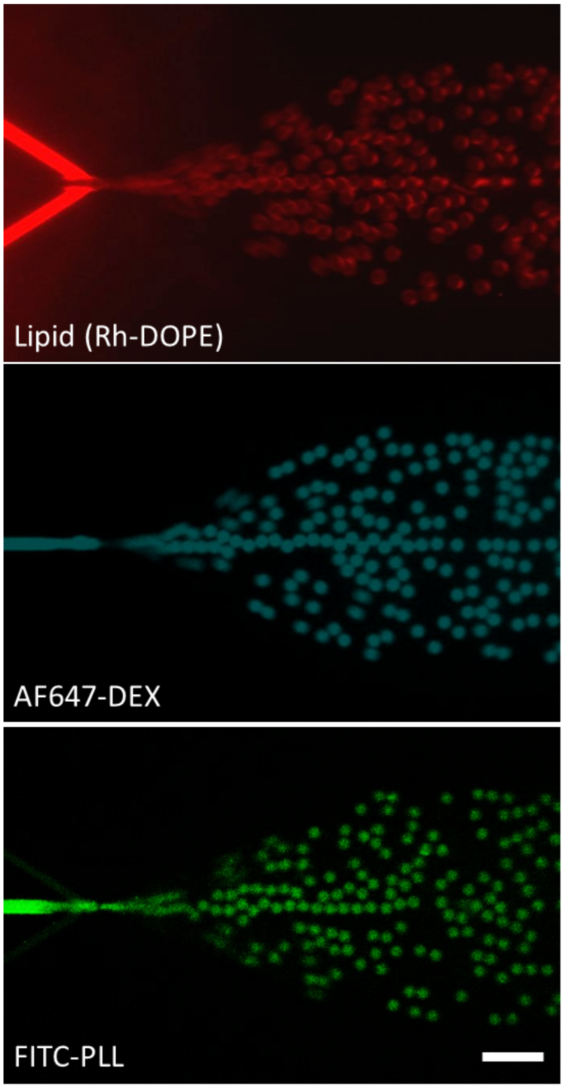

Supporting Figure 4: **Efficient production of monodispersed double emulsions with excellent encapsulation using on-chip OLA technique.** The fluorescent lipid channel shows the formation of double emulsions with octanol pockets, which eventually separate in the post-proudction channel to form liposomes. The DEX and PLL fluorescence show excellent encapsulation and coexistence of these molecules within the formed double emulsions. The lipid composition was 99.9 % DOPC + 0.1% Rh-DOPE (molar ratio) in 1-octanol. The IA solution consisted of 7.3 mM DEX, 8.6 mM PEG, 2.4 mg/ml PLL, 0.8 mM ATP, and 15% v/v glycerol in 15 mM citrate-HCl (pH 4). Scale bar, 50 µm.

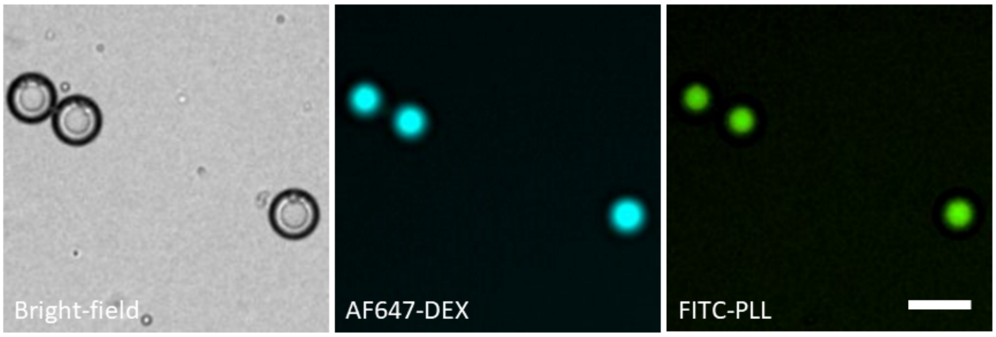

Supporting Figure 5: **Exposing double emulsions to a hypertonic, high-pH buffer with a higher ionic strength (25 mM Tris-HCl) does not lead to coacervation but only SMC formation.** Bright-field and corresponding fluorescence images, captured 2 hours after the trigger. The bright-field image shows a single SMC domain in each double emulsion, as confirmed by the DEX fluorescence. The PLL fluorescence shows complete overlap with DEX fluorescence, but no higher-fluorescence region is present within the SMC domain, indicating complete lack of coacervation. The IA solution consisted of 7.3 mM DEX, 8.6 mM PEG, 2.4 mg/ml PLL, 0.8 mM ATP, and 15% v/v glycerol in 25 mM citrate-HCl (pH 4). The double emulsion suspension was combined with an equal volume of feeding aqueous solution containing 600 mM sucrose and 15% v/v glycerol in 25 mM Tris-HCl (pH 9). Scale bar, 50 µm.

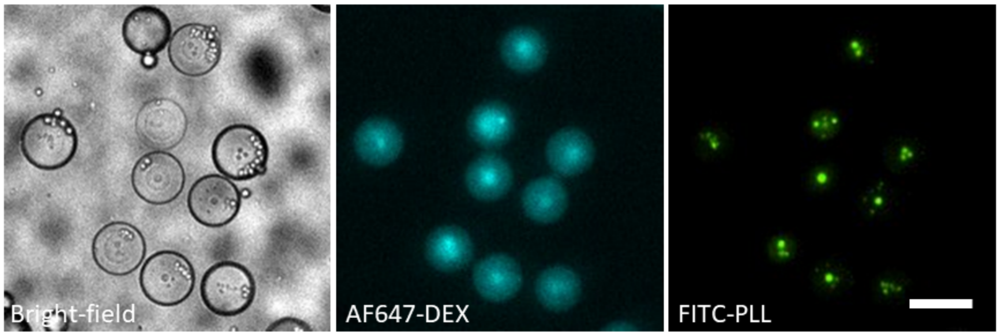

Supporting Figure 6: **Simultaneous SPS and APS triggers form coacervates within SMC domains.** The bright-field image shows a multiphase structure in each double emulsion 2 hours after the hypertonic, high-pH trigger. The largest domain is an SMC, as confirmed by the DEX fluorescence. The SMC domain in turn shows confined coacervates, as confirmed by the corresponding PLL fluorescence. The IA solution consisted of 7.3 mM DEX, 8.6 mM PEG, 2.4 mg/ml PLL, 0.8 mM ATP, and 15% v/v glycerol in 15 mM citrate-HCl (pH 4). The double emulsion suspension was combined with an equal volume of feeding aqueous solution containing 500 mM sucrose and 15% v/v glycerol in 15 mM Tris-HCl (pH 9). Scale bar, 50 µm.

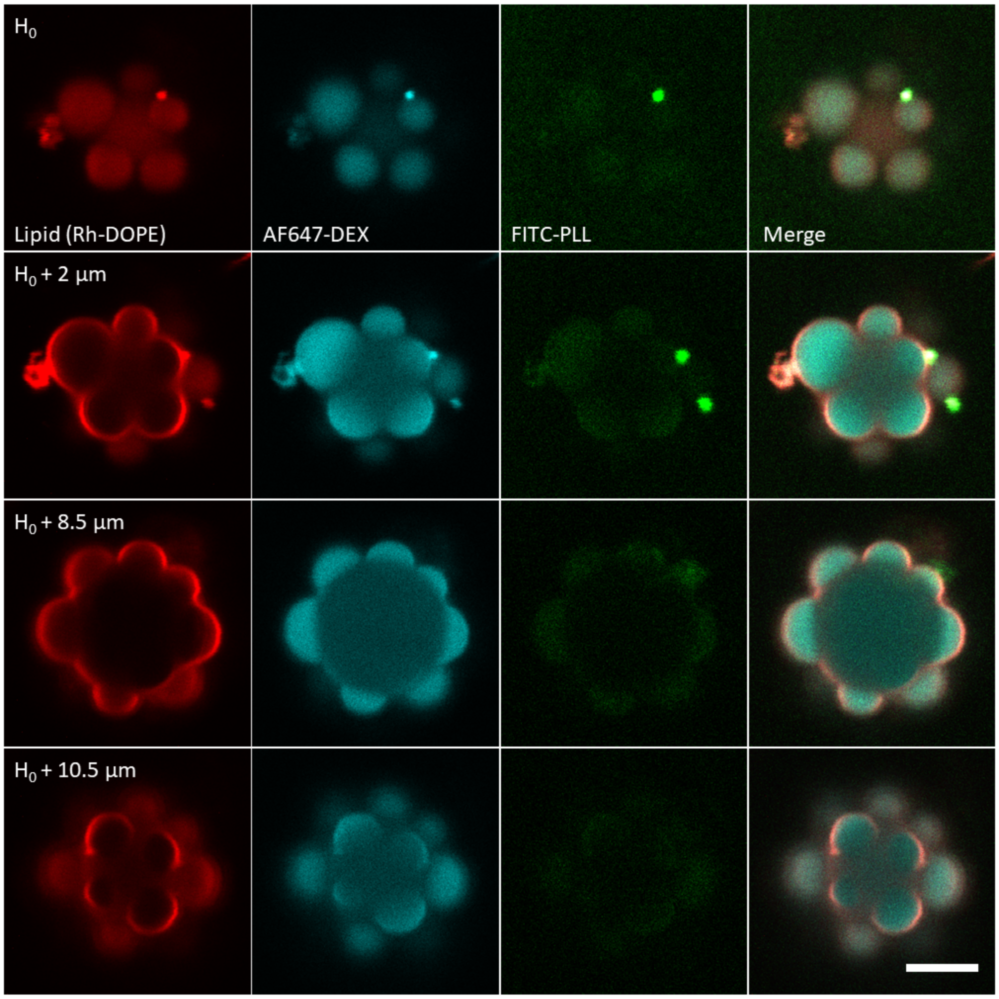

Supporting Figure 7: **Confocal fluorescence images showing the different planes of a 'flower-shaped' liposome with the SMC 'petals' harboring condensates.** The lipid channel shows the pronounced non-spherical morphology of the liposome 2 hours after the hypertonic, high-pH trigger. The DEX fluorescence shows the presence of SMCs that form DEX-rich domains. The PLL fluorescence shows multiple coacervates confined within different SMCs. The lipid composition was 99.9 % DOPC + 0.1% Rh-DOPE (molar ratio). The IA solution consisted of 7.3 mM DEX, 8.6 mM PEG, 2.4 mg/ml PLL, 0.8 mM ATP, and 15% v/v glycerol in 15 mM citrate-HCl (pH 4). The liposome suspension was combined with an equal volume of feeding aqueous solution containing 500 mM sucrose and 15% v/v glycerol in 15 mM Tris-HCl (pH 9). Scale bar, 5 µm.

Supporting movie legends

Supporting Movie 1

Restricted motion of coacervates within MSCs, accentuated by an external fluid flow. We obtained coacervate-in-MSC multi-phase emulsion droplets after mixing the four components under APS and SPS conditions. The coacervates (visualized using FITC-PLL fluorescence and seen as bright green droplets) remain confined within the DEX-rich domains even in presence of an external fluid flow.

Supporting Movie 2

Two-dimensionally restricted movement of PLL-recruited SMCs on the membrane surface. The motions of several membrane-bound SMCs (visualized by FITC-PLL fluorescence, in green) are restricted along the membrane surface (visualized by Rh-DOPE fluorescence, in red).

Supporting Movie 3

Formation of SPS and APS after exposing the liposome to a hypertonic, high-pH buffer. After exposing the liposomes with a hypertonic, high-pH buffer, the liposome (visualized by Rh-DOPE fluorescence, in red) underwent volume reduction, with the extra lipids forming a pocket. Over time, DEX-rich domains (visualized by AF647-DEX fluorescence, in cyan) were formed, exhibited coalescence, and ultimately wetted the membrane. The formed coacervates (visualized by FITC-PLL fluorescence, in green) remained confined within the DEX-rich domains at the membrane.

Supporting Movie 4

3D projection showing the morphology of a liposome 20 minutes after the APS and SPS were triggered. Multiple 'petals' (visualized by AF647-DEX fluorescence, in cyan) could be seen, drastically restructuring the liposome into a 'flower' shape. Some

‘petals’ harbored the formed coacervates (visualized by FITC-PLL fluorescence, in green) as buds on membrane. The SMCs and the buds remained covered by the lipid membrane (visualized by Rh-DOPE fluorescence, in red).

Supporting Movie 5

Confocal fluorescence *z*-stack showing a liposome at different planes after the formation of APS in SMCs. From bottom to up, multiple ‘petals’ (visualized by AF647-DEX fluorescence, in cyan) could be seen, drastically restructuring the liposome into a ‘flower’ shape. The liposomes show different morphologies in different planes due to the random distribution of the petals. Some petals harbored the formed coacervates (visualized by FITC-PLL fluorescence, in green). The lipid membrane is visualized by Rh-DOPE fluorescence, in red.

Supporting Movie 6

Corralled diffusion of coacervates in SMCs at the liposome membrane. The lipid membrane is visualized by Rh-DOPE fluorescence, in red. Due to the separation of the DEX-rich domains (visualized by AF647-DEX fluorescence, in cyan), the coacervates (visualized by FITC-PLL fluorescence, in green) remained isolated and could not come in contact with coacervates residing in other petals. The coacervate trajectories in each petal are random but restricted within corresponding SMCs.

Source legends

Source Code

MATLAB scripts used for image processing and related calculations. All codes used in the figures are listed separately as individual files.

Source Data

Raw data underlying Figures 1-4 in the main article and Supporting Figures 1-2 in Supporting information.
